# Modified use of thoracic and iliac branch endografts to treat an abdominal aortic aneurysm with an unusually narrow neck

**DOI:** 10.1259/bjrcr.20150402

**Published:** 2016-05-05

**Authors:** Edward Norman, Matthew Harling, Alicia Levena Skervin, Celia Riga, Mohamed Khalifa, Richard Gibbs, Mohamad Hamady

**Affiliations:** ^1^ Mersey Foundation School, London, UK; ^2^ South Thames Foundation School, London, UK; ^3^ North West Thames Foundation School, London, UK; ^4^ Section of Vascular Surgery, Division of Surgery and Cancer, Imperial College London, London, UK; ^5^ Interventional Radiology, Imperial College London, London, UK; ^6^ Division of Surgery and Cancer, Imperial College Healthcare NHS Trust, London, UK; ^7^ Department of Interventional Radiology, Imperial College London, London, UK

## Abstract

Abdominal aortic aneurysms with hostile anatomy are a recognized hindrance to the continuing application of endovascular aortic interventions. Narrowed aneurysm necks pose technical difficulties, particularly in the absence of customized endografts. There are multiple suggested approaches to overcome shortened and angulated necks endovascularly; however, none of these address narrowed necks. We present a case where an endograft was used outside of its “instruction for use” by combining the thoracic and iliac branch technologies to overcome this problem. Expanding the use of commercially available endografts for aortic aneurysms with hostile anatomy could have significant practical and financial benefits.

## Summary

Endovascular aneurysm repair (EVAR) is a widely accepted treatment modality for abdominal aortic aneurysm (AAA). The superiority of short-term outcomes for minimally invasive interventions over open surgery has led to recent advances in endovascular technology and techniques.^1^ The “instructions for use” (IFU) of currently available endovascular devices assess the eligibility of the patient in relation to the morphology of the aneurysm. Suitability for EVAR is mostly limited to patient groups that fit within the IFU.

Endografts should be oversized by 10–20% of the native aorta diameter in order to achieve appropriate seal and prevent stent migration.^2^ Oversizing more than 25–30% of the native neck diameter could result in increased incidence of stent folding, migration, neck degeneration and dilatation, and subsequent endoleak.^3–5^ Therefore, the use of EVAR is excluded in patients with an aneurysm neck diameter that is significantly smaller than the smallest available “off-the-shelf” device. We present a modified technique to overcome the challenging anatomical morphology of a narrowed aorta by carefully planned use of a thoracic and iliac branch endograft outside its IFU.

## Case presentation

**Figure 1.**
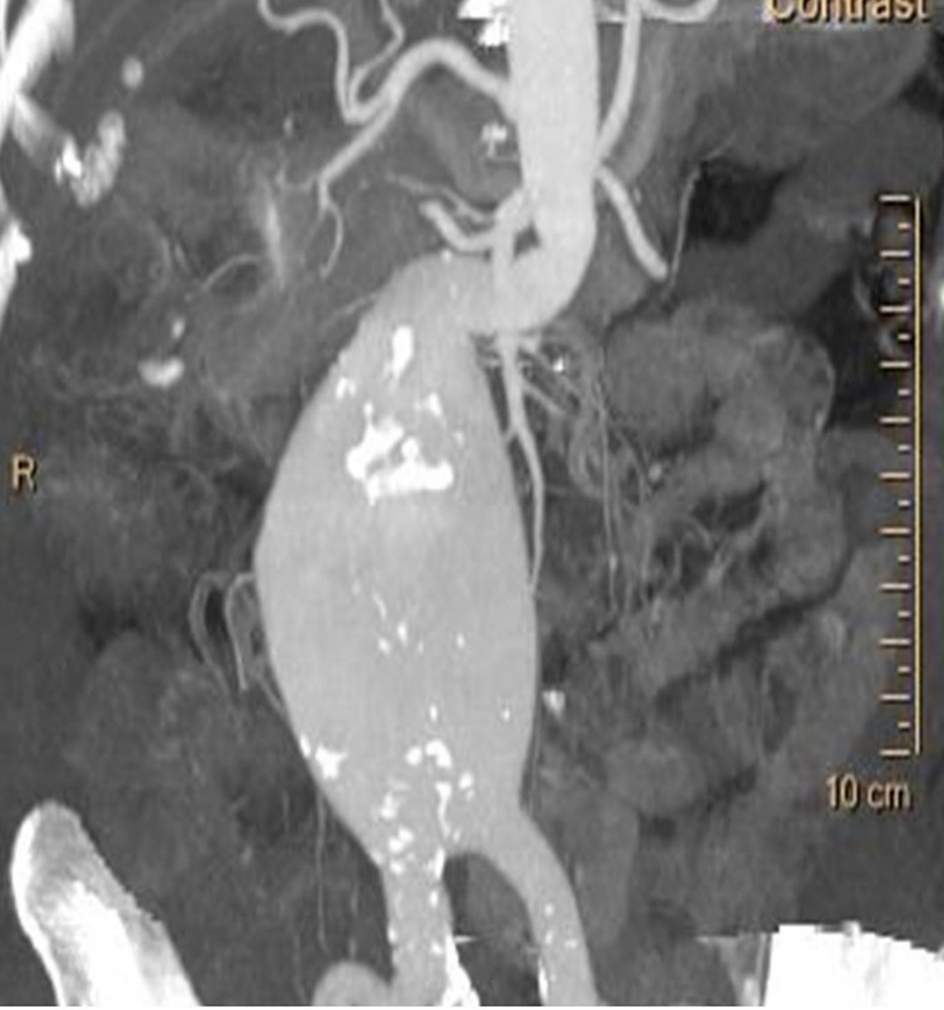
Reconstructed CT angiogram demonstrating the infrarenal abdominal aortic aneurysm and its angulated narrow neck.

An 81-year-old female presented to our tertiary referral centre with non-specific epigastric pain of increasing severity and frequency. Comorbidities were limited to hypertension. The presence of a 67-mm infrarenal AAA extending distally to the aortic bifurcation was confirmed on CT angiography (Figure 1). The aneurysm sac contained no intraluminal thrombus. The neck, however, was extremely angulated with the proximal landing zone measuring 16 mm in diameter. The right common iliac artery was ectatic and measured 16 mm, with the left being of normal calibre and appearance. Both external iliac arteries were found to be tortuous but within normal limits in diameter and measured 7.8 and 7.6 mm on the right and left side, respectively. A stress echocardiogram showed good left ventricular function. Pulmonary function was satisfactory with a forced vital capacity of 125% of predicted and forced expiratory volume in 1 s/forced vital capacity ratio of 84%.

Given the patient’s age and general condition, the surgeon’s assessment stated that open surgery would carry a significant risk of morbidity and mortality. Therefore, EVAR was thought to be the preferred treatment modality, reflecting the advantages of minimally invasive surgery and the patient’s preference over open repair. The significantly narrowed aneurysm neck precluded the use of standard endografts. “Off-the-shelf” abdominal aorta devices have a minimum diameter of 23 mm. For our patient, this entailed 43% oversizing of the native aorta. To overcome the challenging anatomical morphology, we considered a novel endovascular option of deploying a small diameter and short thoracic endograft into the proximal neck of the aneurysm combined with telescopic deployment of a branched iliac device. Consensus was gained to proceed with this approach following discussion at our local multidisciplinary team meeting.

**Figure 2.**
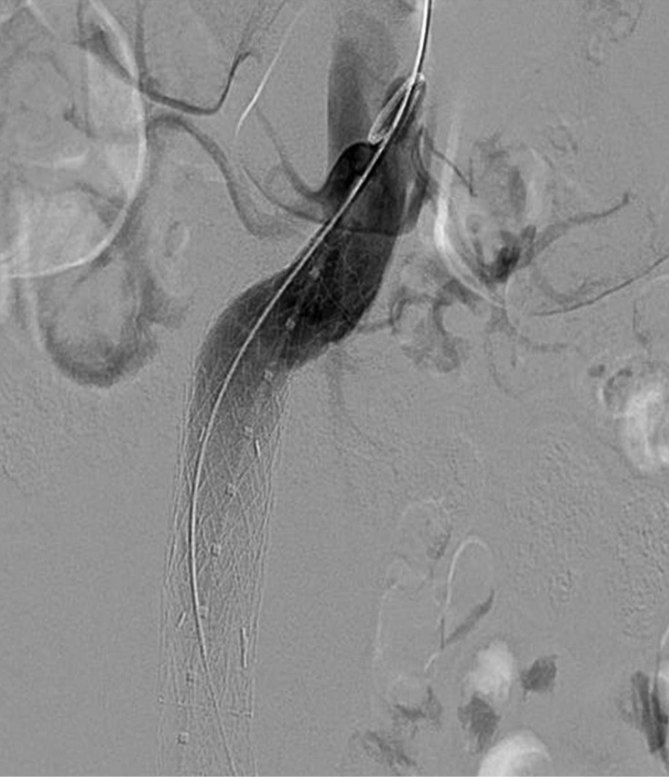
Deployment of the infrarenal thoracic stent into the proximal neck of the abdominal aortic aneurysm.

Intraoperatively, the patient was positioned supine and adequately prepped. Surgical access was obtained *via* bilateral groin cut-downs. Following systemic heparinization (5000 units), a pigtail catheter was introduced into the left common femoral artery through a 9 Fr sheath. Under fluoroscopic guidance, through an introducer sheath (GORE® DrySeal Sheath, W.L. GORE & Associates, Flagstaff, AZ), a 21 × 100 mm conformable thoracic aortic graft (cTAG) thoracic endoprosthesis (W.L. GORE & Associates); 25% oversizing to the aorta) was introduced through the right common femoral artery and deployed just below the level of the renal arteries under fluoroscopic guidance (Figure 2).

**Figure 3.**
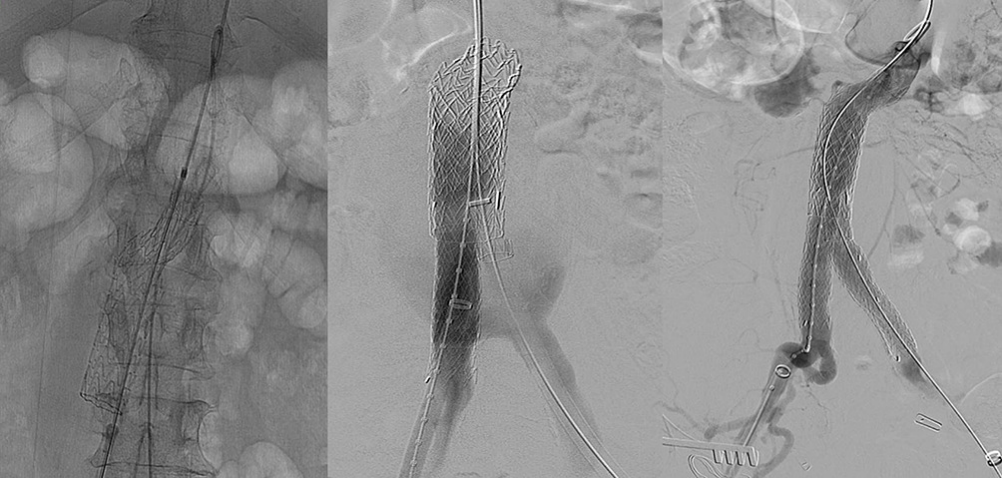
Stepwise deployment of a branched iliac device within the distal aspect of the conformable thoracic aortic graft thoracic endoprosthesis, followed by an extension into the left iliac artery. The Palmaz stent (Cordis, Bridgewater, NJ) within the two stentgrafts is also shown.

**Figure 4.**
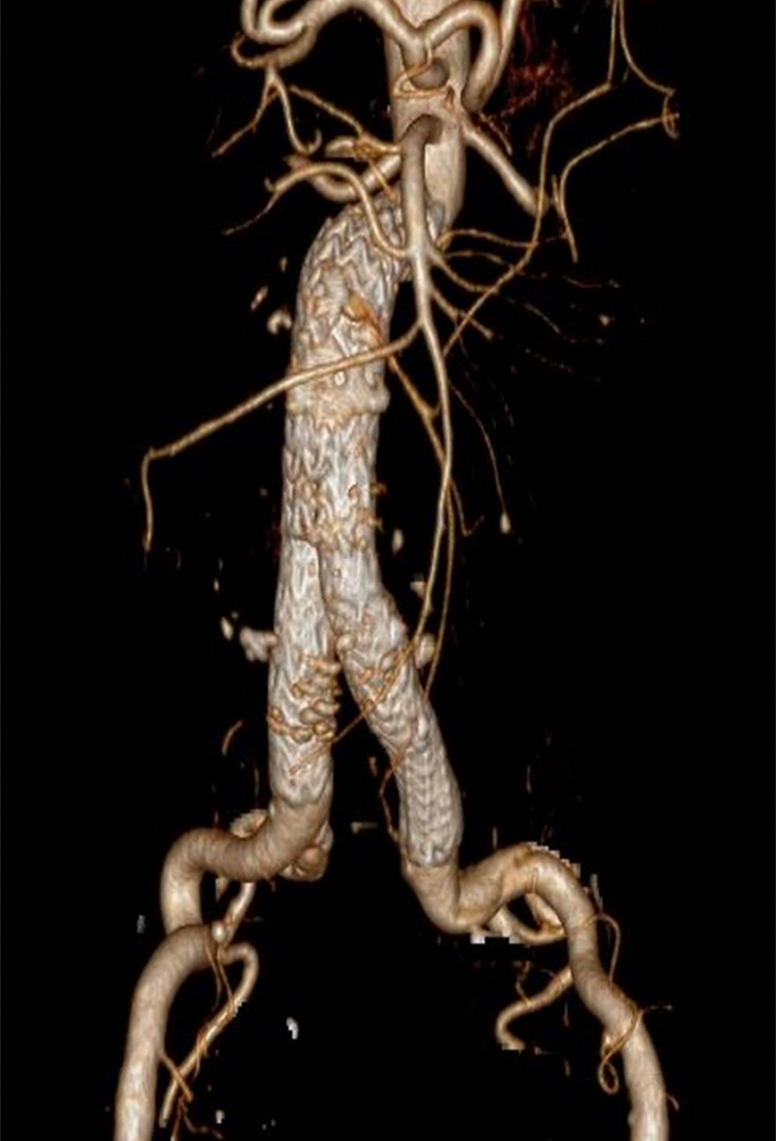
Reconstructed CT angiography demonstrating satisfactory position of the modified stent graft with exclusion of the abdominal aortic aneurysm.

A 14.5 × 100 mm branched iliac device (W.L. GORE & Associates) was then introduced *via* the right common femoral artery and deployed within the distal aspect of the cTAG device, ensuring that there was at least a 30-mm overlap. A 65-cm robotic remotely steerable coaxial catheter system (Hansen Medical, Mountain View, CA) was used to navigate the tortuous iliac anatomy and successfully cannulate the contralateral iliac branch. An 18 × 95 mm extension iliac limb (W.L. GORE & Associates) was then introduced over a stiff wire to extend into the left common iliac artery. The Palmaz XL unmounted stent with an expansion range of 14–25 mm (Cordis, Bridgewater, NJ) was loaded over Omega NV Valvuloplasty balloon catheter (Cook Medical, Bloomington, IN). The stent was partially deployed by manual inflation, followed by full deployment using an inflation device up to 2 atmosphere pressure. A Palmaz stent (Cordis) was positioned overlapping the two endograft devices to enhance the radial forces at the infrarenal portion and prevent future migration (Figures 3 and 4). Completion angiography revealed satisfactory appearance of the composite device with no evidence of an endoleak. There were no immediate postoperative complications.

Postoperatively, the patient made an excellent recovery and was discharged home on day 5. CT angiography at 1 and 6 months demonstrated good position of the infrarenal aortic endograft and exclusion of the aneurysm (Figure 4). A small Type 2 endoleak from the inferior mesenteric artery was noted, which, along with the overall sac size, remained stable at the 6-month scan.

## Discussion

The short-term reduction in morbidity and mortality rates associated with EVAR has led to endovascular interventions being increasingly considered as an attractive alternative to conventional open surgery.^1^ For hostile aneurysm neck anatomy, the IFU of “off-the-shelf” endografts do not recommended their use in complex anatomy, such as more than 65° angulation or small diameter necks that require oversizing by more than 25%.^2^ As anatomical variation is a pertinent factor in this patient cohort, the technical limitations of the currently available endografts restrict their use in anatomically complex cases.

In our tertiary institution, the smallest abdominal aortic stent available was 23 mm in diameter. When oversizing was accounted for, there was no “off-the-shelf” option within the IFU suitable for this case. Thoracic endografts are of a smaller diameter than abdominal devices; this reflects the smaller calibre of the thoracic aorta in young patients. Although licensed for use in thoracic EVAR and trauma, the use of thoracic stents has not been extended to the abdominal aorta until now.

Our case highlights the limitations of available “off-the-shelf” devices for repair of aneurysms with complex anatomy. Outcomes have been found to be poorer in patients with hostile neck anatomy, with higher rates of early and late Type 1 endoleaks and increased requirement for re-interventions reported.^6^ However, EVAR offers a proven benefit to patients who would otherwise be unsuitable for open repair.^7^ This emphasizes the need for innovations and advances in endovascular technologies to overcome difficulties in hostile aneurysms to enhance outcomes and applicability in all patients.

The literature provides a number of alternative approaches to overcome short and angulated neck anatomy.^8–10^ However, none of these addresses small and narrowed necks. The most transferable endovascular approach to a narrowed aortic neck is the use of custom-made devices.^11^ When used routinely, the outcomes have been shown to be very promising.^12^ This bespoke solution, which is advantageous to using “off-the-shelf” devices, is limited by the considerable manufacturing time of up to 8 weeks,^13^ thereby prohibiting its use in emergency situations. In addition, there are financial implications if custom-made devices were to be widely used.^12,13^

An alternative solution would be for institutions to stock a wider range of the current “off-the-shelf” endografts. Having immediate access to a greater number of devices could allow a more comprehensive range of aneurysm anatomy to be routinely treated endovascularly. Practically, however, this is not a feasible option. The number of endovascular devices that would have to be stocked would be too great and economically inefficient.^14^ An interesting proposal from one research group is the formation of a central graft repository providing “next day” delivery.^15^

Our novel approach to addressing small, narrowed neck aneurysms demonstrated that, in the absence of either customized devices or “off-the-shelf” endografts with appropriate IFU, satisfactory outcomes may be achieved by using currently available devices. However, the presence of an experienced team when using equipment outside the IFU is paramount and the procedure should be planned with caution. There are obvious questions over the suitability and safety of such approaches, especially in centres with less experience. Further appraisal of the suitability of devices for a wider range of procedures could have significant practical and financial benefit.

Ultimately, in the absence of customized devices or appropriate “off-the-shelf” endografts, cases of small and narrowed aneurysm necks will continue to pose technical difficulties. We have shown that it is possible to avoid open surgery in these cases with planned and careful extension of the IFU and the innovative use of existing devices.

## Learning points

1. There are still some limitations in the current stent graft technology and more refinements are still needed.
2. Accurate pre-procedure planning is crucial to avoid unnecessary complications.
3. Comprehensive understanding of available technology in various parts of the aorta is helpful to devise innovative solutions in unusual circumstances.

## Consent

Informed written patient consent has been obtained.
